# Second birth intentions and its influencing factors among reproductive-aged women: a cross-sectional study conducted in Shandong Province, China

**DOI:** 10.3389/fpubh.2025.1665360

**Published:** 2025-10-27

**Authors:** Wenhui Cui, Xiuping Guo, Keqing Shi, Mengjun Cao, Zihan Zhou, Hongyan Hao, Ying Zhao, Hongjing Wang, Qiang Wang

**Affiliations:** ^1^College of Public Health, Shandong Second Medical University, Weifang, Shandong, China; ^2^Department of Hospital Infection Management, Qihe People’s Hospital, Dezhou, Shandong, China; ^3^College of Management, Shandong Second Medical University, Weifang, Shandong, China; ^4^Department of Epidemiology, Shandong Second Medical University, Weifang, Shandong, China

**Keywords:** universal two-child policy, reproductive-aged women, second birth intention, influencing factors, fertility policy

## Abstract

**Aims:**

This study investigated second birth intentions and its influencing factors among reproductive-aged women in Shandong Province, China, within the context of the Universal Two-Child Policy (UTCP).

**Background:**

Refining fertility policies and enhancing fertility rates constitute pivotal strategies for China to mitigate the challenges posed by population aging. Understanding fertility intention and its influencing factors is the foundation for refining fertility policies and enhancing fertility rates. As a traditional populous province of China, it has some representativeness to explore the second-birth intentions and their influencing factors of reproductive-aged women in Shandong Province within the context of the UTCP.

**Methods:**

A cross-sectional questionnaire survey was conducted among 2,422 reproductive-aged women (18–45 years) randomly recruited from Shandong Province. Univariate analysis and binary logistic regression analyses were used to identify factors associated with second-birth intentions.

**Results:**

Only 48.02% of respondents expressed willingness to have a second child. The results showed that influencing factors of second birth intentions (*p* < 0.05) included actual fertility timing, impact of household economic status on actual fertility intention, awareness of fertility policy, place of household registration, impact of social and familial expectations on actual fertility intention, marital status, impact of personal career development on actual fertility intention, household size, impact of challenges of childcare on actual fertility intention, self-health status, impact of perceptions of fertility on actual fertility intention and only-child status (ranked by the importance of influencing factors).

**Conclusion:**

Various social, economic and personal factors limit second-birth intentions among reproductive-aged women in Shandong. Targeted policies to reduce childcare burdens, support only-child families and protect women’s health and work rights can foster sustainable fertility intentions.

## 1 Introduction

Fertility intention refers to an individual’s willingness or attitude toward childbearing, a key driver and predictor of fertility behavior and policy (1, 2). To some extent, understanding fertility intention and its influencing factors is the foundation for refining fertility policies and enhancing fertility rates. Therefore, research on fertility intention is essential for low-fertility countries to copy with persistently low fertility challenge, such as China.

Since China established family planning as a fundamental national policy in 1982, the population growth rate has been significantly curbed. However, with the implementation of the family planning policy, negative consequences such as population aging, a shrinking young labor force and a declining birth rate gradually began to surface (3). According to the Fifth National Population Census in 2000, China’s total fertility rate (TFR) among reproductive-aged women was 1.22. By the Sixth National Population Census in 2010, the TFR had declined to 1.18. In both instances, these figures ranked among the lowest total fertility rates in the world at the time (4). To address the problems caused by the family planning policy, policymakers gradually relaxed China’s strict birth control regulations. In 2016, the universal two-child policy (UTCP) was officially implemented nationwide. This policy allowed each couple to have two children to promote more balanced population development (5). Actual birth statistics and previous studies indicate that the universal two-child policy does not take satisfactory long-term effect on enhancing birth rate, despite its short-term impact on enhancing birth rate in 2016–2017 (6–8). Against this backdrop, further optimizing fertility policies and enhancing individual reproductive willingness have emerged as new challenges for the Chinese government. In some extent, comprehending individuals’ fertility intention and its determinants serves as the empirical foundation for formulating effective fertility policy. Thus, it is beneficial for optimizing fertility policies to investigated secondary childbearing intention and influencing factors among reproductive-aged women in the context of UTCP.

Despite this, few studies have focused on the secondary childbearing intention of reproductive-aged women in Shandong Province after the promulgation of UTCP. Shandong represents a typical case in terms of population size, demographic changes and fertility challenges, as one of China’s most populous provinces. Conducting studies in Shandong provides representative insights that can inform both regional and national policy development. According to the Theory of Planned Behavior (TPB), individual intentions to perform a behavior are determined by attitudes, subjective norms and perceived behavioral control, such as having a second child. This theoretical framework has been widely applied in reproductive health studies (2, 9). Therefore, this study aims to assess second-birth intentions among reproductive-aged women in Shandong Province and examine the key demographic, economic, health and policy factors influencing them under the UTCP. The study also aims to provide scientific evidence to support policymakers in optimizing fertility support policies and promoting rational population structure adjustment. It further offers empirical data that may serve as a reference for research in other regions.

## 2 Materials and methods

### 2.1 Definition of participants

The current cross-sectional study was conducted in Shandong Province, China, in 2024. The participants of this study were reproductive-aged women between 18 and 45 years old who were registered residents of Shandong Province. According to international definitions, reproductive-aged women include all women aged 15–49 years, regardless of marital status (10). In China, individuals aged 18 years or older are considered adults (11). Therefore, the lower age limit of 18 years was set to ensure that participants are adults. Women older than 45 face higher childbirth risks, lower fertility and declining ovarian function. According to previous finding, women aged 40–49 have limited contribution to Chinese fertility (12). Based on the above mentioned, the upper age limit of 45 years was set. The study exclusively included women with permanent household registration in Shandong Province, thereby excluding migrant populations without local permanent household registration to ensure sample representativeness and inter-group comparability.

The inclusion criteria: (1) reproductive-aged women (18–45 years old) with household registration in Shandong Province. (2) women participating this study voluntarily. (3) women being able to complete the questionnaire independently. The exclusion criteria: (1) women who were unable or unwilling to participate in the survey. (2) women whose age was below 18 or above 45 years. (3) women without permanent household registration of Shandong Province.

### 2.2 Sample size

In this study, the required sample size was calculated using the standard formula for estimating a single proportion (13).


$$n=\frac{{Z_{\alpha /2}^{2}}^{*}p^{*}(1-p)}{d^{2}}$$

In this formula, *n* is the required sample size, Z_α/2_ is the standard normal value corresponding to the desired confidence level, *p* is the estimated proportion and *d* is the allowable margin of error. A 95% confidence level was adopted (Z_α/2_ = 1.96), and the proportion of fertility intention was conservatively estimated at *p* = 0.5 to maximize the sample size requirement (14). The allowable margin of error (*d*) was set at 0.03. Substituting these values into the formula yielded:


$$n=\frac{1.96^{2}\times 0.5\times (1-0.5)}{0.03^{2}}\approx 1,067$$

The preliminary calculation indicated that the required sample size was 1,067. Considering the design effect due to multistage sampling (Design Effect, DEFF = 2) and an anticipated 10% rate of invalid questionnaires (15), the final target sample size was calculated as follows. Firstly, the adjusted sample size after applying the design effect was.


$$n_{1}=n\times \text{DEFF}$$

In this formula, *n* is the preliminary sample size and DEFF is the design effect. Then, accounting for the expected non-response rate r, the final target sample size was.


$$n_{\text{final}}=\frac{n_{1}}{1-r}$$

Substituting the values from this study (*n* = 1,067, DEFF = 2, *r* = 0.10) gives:


$$\begin{array}{l} n_{1}=1,067\times 2=2,134\\ n_{\text{final}}=\frac{2,134}{1-0.10}=2,371 \end{array}$$

Thus, the final target sample size was 2,371.

In this study, a total of 2,700 questionnaires were distributed. After excluding those with logical inconsistencies, missing data, or non-compliance with the inclusion criteria, 2,422 valid questionnaires were obtained, meeting the analysis requirements.

### 2.3 Sampling method

This study was a cross-sectional study. The multi-stage sampling method was used to randomly sample participants.

Firstly, the 16 cities of Shandong Province exhibit significant demographic heterogeneity, attributed to divergent social, economic and cultural conditions. Consequently, they were stratified into three distinct clusters during phase one, with classification criteria integrating socioeconomic and cultural dimensions. Secondly, three cities were randomly selected from each distinct clusters as sample cities. The random sampling methodology ensures fairness in city selection to some extent, as each city within a given group has an equivalent chance of participation, which strengthens the overall sample validity. Thirdly, one county or district (county-level city) was randomly selected from each of the selected sample cities as sample county or district; Fourthly, one subdistrict and one township were further randomly selected as sample subdistrict and township from each county-level sample county or district. Fifthly, one urban residential community was randomly selected from the sample subdistrict and one rural village was randomly selected from the sample township. The sample of reproductive-aged women was composed by individuals of sample community or village who met the inclusion and exclusion criteria. At each stage, the number of urban communities and rural villages was proportionally allocated according to the actual number of reproductive-aged women in each area. This approach was intended to minimize overrepresentation of urban participants and ensure representativeness of both urban and rural populations. Ultimately, a sample size of 2,422 was obtained.

By using proportional random selection at each stage and a relatively large sample size, it ensured that the final sample was representative, random and reliable.

### 2.4 Questionnaire survey

In December 2024, an online questionnaire survey was conducted among reproductive-aged women selected from various prefecture-level cities in Shandong Province. The core content of the questionnaire was the willingness to have a second child under the current fertility policy context. Participants were divided into two groups based on “willing to have a second child” and “unwilling to have a second child.” It aimed to explore the potential mechanisms influencing the actual intention to have a second child. To systematically investigate the key factors influencing the fertility intentions, the questionnaire covered variables from multiple dimensions, including: age, residence, place of household registration, educational level, occupation, marital status, type of original family, only-child status, long-term migration status, household size, monthly household income, monthly household expenditure, self-health status, family health status, awareness of fertility policy, childcare before school enrollment, actual fertility timing, impact of household economic status on actual fertility intention, impact of social and familial expectations on actual fertility intention, impact of perceptions of fertility on actual fertility intention, impact of challenges of childcare on actual fertility intention, impact of fertility policy on actual fertility intention, impact of child upbringing on actual fertility intention, impact of personal career development on actual fertility intention. The selection of these variables was guided by TPB (16). This theory suggests that individual behavior is influenced by three core components: attitude toward the behavior, subjective norms, and perceived behavioral control. Based on this theoretical framework, the questionnaire variables were classified into the three TPB components as follows. Variables reflecting attitude toward the behavior included self-health status, family health status, awareness of fertility policy, perceptions of fertility, impact of perceptions of fertility on actual fertility intention, and impact of fertility policy on actual fertility intention, capturing personal evaluations and beliefs about having a second child. Variables representing subjective norms included impact of social and familial expectations, type of original family, only-child status, and impact of social and familial expectations on actual fertility intention, reflecting perceived social pressures and family expectations. Variables representing perceived behavioral control included household economic status, childcare before school enrollment, challenges of childcare, impact of household economic status on actual fertility intention, impact of challenges of childcare on actual fertility intention, impact of child upbringing on actual fertility intention, personal career development, and impact of personal career development on actual fertility intention, capturing perceived ease or difficulty in having a second child given personal and environmental constraints. A questionnaire was designed using a five-point Likert scale to analyze the influence of seven factors on the willingness of reproductive-aged women to have a second child. Each item within the dimensions was scored from 1 to 5, with 1 = “no impact,” 2 = “small impact,” 3 = “moderate impact,” 4 = “large impact” and 5 = “very large impact.” Higher scores for each dimension indicated a greater influence of the factor on second-child fertility intentions. The detailed list of all items and their corresponding dimensions was provided in Supplementary Table 1. These factors were: impact of household economic status on actual fertility intention, impact of social and familial expectations on actual fertility intention, impact of perceptions of fertility on actual fertility intention, impact of challenges of childcare on actual fertility intention, impact of fertility policy on actual fertility intention, impact of child upbringing on actual fertility intention and impact of personal career development on actual fertility intention. The questionnaire used in this study demonstrated high reliability and validity. Before the formal survey, a pilot study was conducted to pre-test the questionnaire. A small sample of 200 women aged 18–45 comprising female workers and students completed the questionnaire online. This pilot survey helped correct potential issues and refined the questionnaire to improve comprehensiveness. In the formal survey, the Kaiser–Meyer–Olkin (KMO) value was 0.940 and Bartlett’s test of sphericity was significant (*p* < 0.001), indicating that the variables were suitable for factor analysis. Cronbach’s alpha coefficient was 0.955, demonstrating excellent internal consistency.

### 2.5 Statistical analysis

The collected questionnaires were imported into the R 4.3.1 statistical software to establish a database. Descriptive analyses were conducted on the qualified data to summarize the sociodemographic characteristics, actual fertility status and other basic information. For quantitative data with a normal distribution, the mean ± standard deviation was used for presentation; otherwise, the median (interquartile range) was reported. If the quantitative data were normally distributed with homogeneity of variance, comparisons between two or more groups were conducted using *t*-tests or analysis of variance (ANOVA); otherwise, the rank-sum test was used. Qualitative data were expressed as frequency (percentage) and comparisons between groups were conducted using the chi-square test. Logistic regression analysis was used to explore the factors influencing of second birth intention among reproductive-aged women. Given the proportional multi-stage sampling design and large sample size, unweighted regression analyses were considered appropriate for estimating associations between variables. Standard errors were calculated using conventional methods. The dominance analysis was applied to quantify the relative importance of each factor influencing the willingness to have a second child (17, 18). All statistical analyses were conducted using R version 4.3.1. All reported *p*-values were two-sided and statistical significance was defined as *p* < 0.05.

### 2.6 Quality control

Firstly, participants were selected based on the principle of random sampling to minimize selection bias as much as possible. Secondly, efforts were made to enhance publicity to increase participants’ engagement, thereby reducing nonresponse rates and biases caused by subjective reporting errors.

### 2.7 Ethics approval

The procedures followed by the Institute are in line with the ethical standards of the Declaration of Helsinki (1964, most recently revised in 2013) established by the World Medical Association. And this study was approved by the Ethics Review Committee of Shandong Second Medical University (No. 2023YX-139).

## 3 Results

### 3.1 Univariate analysis of factors influencing the willingness of reproductive-aged women to have a second child

The results of Supplementary Table 2 showed that significant statistical differences exist in the willingness of reproductive-aged women.

The age of participants who were willing to have a second child was significantly higher than that of participants who were unwilling (χ^2^ = 93.08, *p* < 0.001). When pairwise comparisons were made between any two age groups for fertility willingness, the distribution differences were also statistically significant (*p* < 0.017).

Significant differences were observed in the willingness to have a second child across place of household registration (χ^2^ = 71.90, *p* < 0.001). In any pairwise comparison of places of household registration, all differences were statistically significant (*p* < 0.008) except for those between “Jiaodong Peninsula” and “Central Shandong,” and between “Northwestern Shandong” and “Southern Shandong,” which were not statistically significant.

Education level was significantly associated with second-child intention (χ^2^ = 41.21, *p* < 0.001). In any pairwise comparison of educational level, participants with “Junior high school or below” had significantly higher willingness to have a second child than those with “College/Bachelor’s degree” or “Postgraduate and above,” and participants with “High school/Vocational/Technical secondary school” had significantly higher willingness than those with “College/Bachelor’s degree” (*p* < 0.008).

Marital status significantly influenced second-child intention (χ^2^ = 121.90, *p* < 0.001). In any pairwise comparison of marital status, participants with “Married with no children” and “Married with children” had significantly higher willingness to have a second child than those with “Unmarried” (*p* < 0.008).

Type of original family was associated with second-child intention (χ^2^ = 44.26, *p* < 0.001). In any pairwise comparison of type of original family, participants with “Backbone family” had significantly higher willingness to have a second child than those with “Nuclear family” (*p* < 0.005).

Occupation significantly affected second-child intention (χ^2^ = 110.50, *p* < 0.001). In any pairwise comparison of occupation, participants with “Civil servant, public institution or state-owned enterprise personnel” and “Individual household,” “Migrant worker,” “Peasant,” “Freelance work,” “Other practitioners” and “Unemployed” had significantly higher willingness to have a second child than those with “Student” (*p* < 0.001).

Household size was significantly associated with second-child intention (χ^2^ = 63.34, *p* < 0.001). In any pairwise comparison of household size, participants with “5–6” members had significantly higher willingness to have a second child than those with “1–2” members and “3–4” members (*p* < 0.008).

Self-health status and family health status were both significantly associated with second-child intention (self-health: χ^2^ = 19.17, *p* < 0.001; family health: χ^2^ = 16.59, *p* < 0.001). In any pairwise comparison of self-health status and family health status, participants with “Very healthy, no illness” and “Healthy, occasional minor illness” had significantly higher willingness to have a second child than those with “Weak and prone to illness” (*p* < 0.017).

Awareness of fertility policy was significantly associated with second-child intention (χ^2^ = 46.89, *p* < 0.001). In any pairwise comparison of awareness of fertility policy, participants with “Somewhat familiar” and “Quite familiar” had significantly higher willingness to have a second child than those with “Not familiar” (*p* < 0.017). Meanwhile, participants with “Somewhat familiar” had significantly higher willingness to have a second child than those with “Quite familiar” (*p* < 0.017).

Actual fertility timing was also significantly associated with second-child intention (χ^2^ = 44.30, *p* < 0.001). In any pairwise comparison of actual fertility timing, participants with “Within one year” and “One to two years” had significantly higher willingness to have a second child than those with “Three to four years” (*p* < 0.005). Meanwhile, participants with “Let nature take its course” had significantly higher willingness to have a second child than those with “Three to four years” (*p* < 0.005).

### 3.2 Logistic regression analysis of influencing factors of second-birth intentions among reproductive-aged women

#### 3.2.1 Collinearity test of independent variables

In this study, the Variance Inflation Factor (VIF) values between the variables ranged from 1.019 to 1.745, which meant that it was well below the critical threshold of 10. Thus, there was no multicollinearity issue among the independent variables examined (Supplementary Table 3). This result met the requirement for collinearity in logistic regression analysis.

#### 3.2.2 Binary logistic regression analysis

The results showed that place of household registration significantly affected the willingness of reproductive-aged women to have a second child. Compared with women of the Jiaodong Peninsula, women of northwestern Shandong were about 2.2 times more probability to have a second child (OR = 2.178, 95% CI: 1.535–3.098, *p* < 0.001). Compared to unmarried women, those who were married with children were about 0.4 times as likely to have a second child (OR = 0.411, 95% CI: 0.241–0.700, *p* = 0.001). Compared with women who were only children, those who were not only children were about 1.3 times more probability to intend a second birth (OR = 1.325, 95% CI: 1.046–1.679, *p* = 0.020). Women with 5–6 family members were about 2.2 times more likely to have a second child compared to those with 1–2 family members (OR = 2.178, 95% CI: 1.341–3.553, *p* = 0.002). Compared to women who considered themselves “very healthy, no illness,” those who considered themselves “weak and prone to illness” were about 0.53 times as likely to intend a second child (OR = 0.527, 95% CI: 0.284–0.958, *p* = 0.038). Regarding awareness of fertility policies, those who “somewhat familiar” were about 1.6 times more probability to intend a second birth (OR = 1.616, 95% CI: 1.225–2.136, *p* = 0.001) and those who were “quite familiar” were about 1.8 times (OR = 1.770, 95% CI: 1.351–2.323, *p* < 0.001) more probability to have a second child compared to those who “not familiar.” In terms of actual fertility timing, compared with women who had their first child “within one year” after marriage, those who had their first child “one to two years” after marriage were only 0.5 times as likely to intend a second child (OR = 0.517, 95% CI: 0.294–0.884, *p* = 0.018). Those who had their first child “three to four years” after marriage were 0.34 times as likely (OR = 0.335, 95% CI: 0.183–0.600, *p* < 0.001), and those who had their first child “five years or more” after marriage were 0.36 times as likely (OR = 0.361, 95% CI: 0.140–0.914, *p* = 0.033). Impact of household economic status on actual fertility intention (OR = 0.946, 95% CI: 0.932–0.959, *p* < 0.001), impact of personal career development on actual fertility intention (OR = 0.896, 95% CI: 0.859–0.934, *p* < 0.001), and impact of challenges of childcare on actual fertility intention (OR = 0.927, 95% CI: 0.893–0.962, *p* < 0.001) significantly reduced the willingness to have a second child. Specifically, for each one-point increase in impact of household economic status on actual fertility intention, the odds of intending a second child were 0.946 times as high. For each one-point increase in impact of personal career development on actual fertility intention, the odds of intending a second child were 0.896 times as high. For each one-point increase in impact of challenges of childcare on actual fertility intention, the odds of intending a second child were 0.927 times as high. Impact of social and familial expectations on actual fertility intention (OR = 1.069, 95% CI: 1.045–1.095, *p* < 0.001) and impact of perceptions of fertility on actual fertility intention (OR = 1.028, 95% CI: 1.005–1.051, *p* = 0.015) significantly increased the willingness to have a second child. Specifically, for each one-point increase in impact of social and familial expectations on actual fertility intention, the odds of intending a second child were 1.069 times as high. For each one-point increase in impact of perceptions of fertility on actual fertility intention, the odds of intending a second child were 1.028 times as high. These results confirmed that regional, familial, health, economic and attitudinal factors independently determined second-birth intentions in this population. The specific results were shown in Table 1.

**Table 1:** Results of binary logistic regression.

Variables	Group	*β*	*SE*	*P*	OR (95%*CI* of OR)
Place of household registration (Reference: Jiaodong Peninsula)	Northwestern Shandong	0.778	0.179	<0.001	2.178 (1.535, 3.098)
	Central Shandong	0.260	0.161	0.106	1.298 (0.947, 1.782)
	Southern Shandong	0.247	0.170	0.147	1.280 (0.917, 1.789)
Marital Status (Reference: Unmarried)	Married with no children	−0.560	0.307	0.068	0.571 (0.313, 1.042)
	Married with children	−0.889	0.272	0.001	0.411 (0.241, 0.700)
	Widowed/divorced	0.261	0.548	0.633	1.299 (0.442, 3.860)
Only-child status (Reference: Yes)	No	0.282	0.121	0.020	1.325 (1.046, 1.679)
Household size (Reference: 1–2)	3–4	0.205	0.232	0.376	1.228 (0.781, 1.938)
	5–6	0.778	0.248	0.002	2.178 (1.341, 3.553)
	≥7	−0.230	0.478	0.630	0.794 (0.309, 2.022)
Self-health status (Reference: Very healthy, no illness)	Healthy, occasional minor illness	0.095	0.147	0.518	1.099 (0.825, 1.466)
	Weak and prone to illness	−0.641	0.309	0.038	0.527 (0.284, 0.958)

Variables	Group	*β*	*SE*	*P*	OR (95%*CI* of OR)
Awareness of fertility policy (Reference: Not familiar)	Somewhat familiar	0.480	0.142	0.001	1.616 (1.225, 2.136)
	Quite familiar	0.571	0.138	<0.001	1.770 (1.351, 2.323)
Actual fertility timing (Reference: Within one year)	One to two years	−0.660	0.280	0.018	0.517 (0.294, 0.884)
	Three to four years	−1.093	0.303	<0.001	0.335 (0.183, 0.600)
	Five years or more	−1.018	0.478	0.033	0.361 (0.140, 0.914)
	Let nature take its course	−0.513	0.303	0.090	0.598 (0.327, 1.072)
Impact of household economic status on actual fertility intention	–	−0.056	0.007	<0.001	0.946 (0.932, 0.959)
Impact of social and familial expectations on actual fertility intention	–	0.067	0.012	<0.001	1.069 (1.045, 1.095)
Impact of perceptions of fertility on actual fertility intention	–	0.028	0.011	0.015	1.028 (1.005, 1.051)
Impact of personal career development on actual fertility intention	–	−0.110	0.021	<0.001	0.896 (0.859, 0.934)
Impact of challenges of childcare on actual fertility intention	–	−0.076	0.019	<0.001	0.927 (0.893, 0.962)

### 3.3 Dominance analysis of influencing factors

Supplementary Table 4 showed that the five most important factors influencing the willingness to have a second child were as follows: actual fertility timing, the impact of household economic status on fertility intention, awareness of fertility policy, place of household registration and social and familial expectations.

## 4 Discussion

This study found that only 48.02% of reproductive-aged women expressed their willingness to have a second child. Besides, the results showed that influencing factors of second birth intentions included actual fertility timing, impact of household economic status on actual fertility intention, awareness of fertility policy, place of household registration, impact of social and familial expectations on actual fertility intention, marital status, impact of personal career development on actual fertility intention, household size, impact of challenges of childcare on actual fertility intention, self-health status, impact of perceptions of fertility on actual fertility intention and only-child status (ranked by the importance of influencing factors).

This study found that only 48.02% of reproductive-aged women expressed their willingness to have a second child. Comparative data showed similarly low levels of fertility intention in low fertility countries, such as South Korea, Japan and some European nations (19–21). The consistency across different contexts suggested that broader socioeconomic factors may have influenced fertility decisions. Economic pressure, career demands and social expectations were probability key contributors. This study highlighted the challenge of increasing second-child fertility in low-fertility settings. These factors may discourage childbearing not only by raising financial costs but also by influencing the perceived opportunity cost of raising children. For example, demanding work schedules and career insecurity could reduce couples’ available time and energy for parenting (22). In addition, social expectations of women’s dual role in employment and family life may contribute to psychological stress and practical pressures (23). In such circumstances, fertility decisions tend to be shaped by concerns over work-life balance and gender role expectations, in addition to household income.

### 4.1 Actual timing of fertility after marriage

The actual fertility timing after marriage showed significant association with the second birth intentions. Compared with women who had their first child within 1 year of marriage, those who delayed childbirth to later years were progressively less likely to intend a second child. Delaying the first childbirth may affect women’s reproductive capacity and their ability to have additional children (24). The optimal physiological window for reproduction often conflicted with pursuing higher education or career development. The decision to postpone childbearing is shaped by both subjective perceptions and objective circumstances. Factors such as socio-economic status, career aspirations and psychological influences all play a significant role in this choice (25). In low-fertility countries such as South Korea Japan and European nations (26, 27), delayed childbearing due to socio-economic, educational and cultural factors has contributed to lower fertility rates. These trends underscore the need to consider fertility timing in policy design and provide targeted support to couples.

### 4.2 Household economic status

The greater the impact of household economic status on fertility intentions, the lower the likelihood of intending a second child. Economic constraints can deter couples from having more children including high living, housing, education and healthcare costs. When economic status strongly influences fertility decisions, the need to maintain financial stability may outweigh the desire for additional children. This effect is especially pronounced in contexts with high economic insecurity. Studies have indicated that couples with higher incomes are more likely to intend to have a second child. They feel better able to manage the financial responsibilities associated with an additional child. In contrast, couples facing economic difficulties often delay or forgo having more children due to perceived financial burdens (28). This aligns with trends observed in other low-fertility countries, where economic factors heavily influence second-birth intentions. In Japan, the high cost of raising children, particularly expenditures on pre-tertiary education, has been identified as a significant barrier to increasing fertility rates. Households with greater financial resources are more likely to have multiple children, whereas those facing economic constraints often choose for smaller families (27). In some low-fertility European countries, higher household income and a smaller share of income spent on basic necessities are associated with stronger intentions to have a second child (29). Economic stability gives couples more confidence to support additional children and thus shapes their fertility intentions. This highlights the importance of economic factors in fertility decisions. Policies that reduce financial burdens may help increase second-birth intentions and curb declining fertility rates, such as financial incentives, affordable childcare and housing support.

### 4.3 Awareness of fertility policies

People who were familiar with fertility policies were more likely to intend having a second child than those who were less or not familiar. Greater familiarity with fertility policies may increase second-birth intentions by reducing cost uncertainty and strengthening perceived institutional support. Awareness of childcare subsidies, parental leave and other benefits helps couples plan more confidently. Lack of information may cause them to overestimate burdens. Similar patterns are seen in Japan. Limited fertility knowledge delays childbearing, whereas greater knowledge shifts attitudes and intentions (30, 31). Enhancing public understanding and communication of fertility policies could therefore be a promising strategy to raise second-birth intentions in low-fertility countries.

### 4.4 Regional differences

This study found regional differences in fertility intentions, with women in Northwestern Shandong more willing to have a second child than those in the Jiaodong Peninsula. A number of explanations may help clarify regional differences. Firstly, as a relatively developed coastal region, the Jiaodong Peninsula has higher living costs, education expenses and housing burdens (32). In contrast, Northwestern Shandong has a slower pace of life and a higher proportion of rural households with more traditional family structures (33). Secondly, Women in the Jiaodong Peninsula also tend to have higher education levels and greater career development opportunities, which may influence their fertility plans (28, 34). Meanwhile, Northwestern Shandong may place greater emphasis on family support and grassroots policy promotion (35). Similar patterns have been observed internationally. Urban women in Japan, South Korea and several European low-fertility countries tend to show lower fertility intentions than rural women. This pattern has been linked to higher housing costs, greater career competition and challenges in balancing work and family (19, 36, 37). By contrast, rural women generally experience fewer career constraints and stronger family support (38). These parallels suggest that the regional differences observed in Shandong may reflect broader socio-economic and cultural determinants of fertility behavior.

### 4.5 Family composition and sibling status

Women from non-only-child families were more likely to have a second child than only-child women, consistent with previous research in China (39). Possible explanations for this difference include perceptions of eldercare pressure and differences in educational level (40). Only-child women having no siblings to share family responsibilities face greater eldercare pressures and may be less willing to have a second child (41, 42). Secondly, only-child group are generally more highly educated (41). Women with higher education are more likely to consider how childbearing affects their career and quality of life. Under limited economic conditions, this may reduce their willingness to have a second child (43). In contrast, women from non-only-child families have siblings to share future eldercare responsibilities (44). This makes them more accepting of larger families and more positive toward having a second child. This phenomenon is not unique to China. Studies in Japan and South Korea have reported that intergenerational care obligations and family-structure factors influence women’s fertility intentions. Research from several European low-fertility countries has also linked intergenerational support concerns, childcare costs and delayed childbearing to differences in parity progression (45). These findings support that family composition and perceived intergenerational responsibilities influence second-child fertility intentions across cultures.

## 5 Study strengths, limitations and policy implications

By presenting the study’s strengths, limitations and policy implications, this study provided a balanced view of its contributions and constraints.

This study had several strengths. Firstly, it included a large sample size of 2,422 valid responses which enhanced the statistical power and reliability of the findings. Secondly, a multi-stage sampling design was employed, ensuring representativeness across different regions and communities within Shandong Province. Thirdly, the questionnaire showed high consistency and validity with a Cronbach’s alpha of 0.955 and a KMO of 0.940. Finally, the study had strong policy relevance and provided evidence to inform fertility-related policies in China, particularly the two-child policy.

However, there were some limitations to this study. Firstly, this study was a cross-sectional study conducted at a specific point in time and lacked longitudinal data to reflect changes over time. Thus, the relationships among significantly valuable independent variables and dependent variable can only be interpreted as statistical correlation rather than causal correlation. Secondly, this study was conducted only in Shandong Province. Although the province has some representativeness, regional differences in economic development, fertility policies and social norms across China may limit the generalizability of the findings. Thirdly, the representativeness of the sample was limited. The occupational types of the respondents were relatively concentrated. Therefore, caution is needed when generalizing the findings to other occupational groups. Fourthly, the data were collected through self-administered online questionnaires, which may introduce self-reporting bias. In addition, limitations inherent to online survey administration may affect the accuracy of the responses, such as digital access disparities. Fifthly, cultural values and social desirability may have influenced how participants reported their fertility intentions. Sixthly, sampling weights were not applied in the analysis, which may have led to minor imbalances in the representation of certain subgroups. Future studies could consider applying appropriate weighting to further enhance generalizability. Finally, potential endogeneity between women’s career development and fertility intentions could not be ruled out. Further clarification of this relationship would require longitudinal or instrumental-variable analyses.

To effectively implement the two-child policy, this study offers recommendations to guide policymakers with scientific evidence and practical advice. Firstly, for actual fertility timing, policymakers should provide flexible fertility support measures. These include extended maternity leave, accessible assisted reproductive services for older women and information campaigns to help couples plan childbirth timing. Secondly, regarding household economic status, targeted financial and welfare support should be increased for families with lower economic status. This should include direct subsidies, childcare cost reductions and preferential housing policies to ease the economic burden of having a second child. Thirdly, for awareness of fertility policy, dissemination and clarity of fertility-related policies should be enhanced through multiple channels such as media, workplace information sessions. This will help couples fully understand the available benefits and support for second-child births. Fourthly, in terms of place of household registration, policy benefits should be adjusted to reduce disparities between urban and rural regions and across registration types. This ensures equal access to fertility-related support services. Finally, for social and familial expectations, a supportive social climate should be fostered. Community education, workplace-family balance initiatives and public campaigns can help reshape traditional or negative family expectations.

## 6 Conclusion

The willingness of reproductive-aged women in Shandong Province to have a second child needs to be improved. Their fertility intentions are influenced by multiple factors, including individual, familial and socio-cultural determinants. Based on these findings, relevant authorities should consider interventions to improve the physical and mental well-being of reproductive-aged women. Measures should also be taken to optimize female employees’ work rights. These actions could help promote shared responsibilities among government, society, and families and support rational and sustainable fertility intentions.

## Data Availability

The original contributions presented in the study are included in the article/Supplementary material, further inquiries can be directed to the corresponding authors.
