# Supplementary material for: Second birth intentions and its influencing factors among reproductive-aged women: a cross-sectional study conducted in Shandong Province, China
Source: Front Public Health. 2025 Oct 27;13:1665360. doi: 10.3389/fpubh.2025.1665360 (PMC12597926; doi:10.3389/fpubh.2025.1665360)
Supplement: Supplementary file 1 [file Table_1.docx]

| Supplementary Table 1 Details of Questionnaire Items and Dimensional Indicators | | |
| --- | --- | --- |
| Dimensional indicators | Options | Item |
| Impact of household economic status on actual fertility intention | Q31-1 | Does your household’s economic condition affect your decision to have two children? |
|  | Q31-2 | Do the expenses of supporting older adults affect your decision to have two children? |
|  | Q31-3 | Do the expenses of raising two children affect your decision to have two children? |
|  | Q31-4 | Do the expenses incurred during the preschool stage of two children affect your decision to have two children? |
|  | Q31-5 | Do the expenses incurred during the primary, junior high and senior high school stages of two children affect your decision to have two children? |
|  | Q31-6 | Do the expenses incurred during the university stage of two children affect your decision to have two children? |
|  | Q31-7 | Does the additional housing cost or pressure resulting from having two children affect your decision to have two children? |
|  | Q31-8 | Do the future housing expenses of two children affect your decision to have two children? |
|  | Q31-9 | Do the future marriage expenses of two children affect your decision to have two children? |
| Impact of social and familial expectations on actual fertility intention | Q33-1 | Does your husband’s expectation of having a second child affect your decision to have two children? |
|  | Q33-2 | Do your parents-in-law’s expectations of having a second child affect your decision to have two children? |
|  | Q33-3 | Do your parents’ expectations of having a second child affect your decision to have two children? |

| Supplementary Table 1 (continued) |  |  |
| --- | --- | --- |
| Dimensional indicators | Options | Item |
|  | Q33-4 | Do your colleagues’, relatives’, neighbors’ and friends’ attitudes toward having a second child affect your decision to have two children? |
|  | Q33-5 | Does society’s expectation of having a second child affect your decision to have two children? |
| Impact of perceptions of fertility on actual fertility intention | Q35-1 | Does the traditional fertility view of “raising sons for old-age support” influence your decision to have two children? |
|  | Q35-2 | Does the traditional fertility view of “continuing the family line” influence your decision to have two children? |
|  | Q35-3 | Does the traditional fertility view of “the more children, the greater the blessing” influence your decision to have two children? |
|  | Q35-4 | Do emerging parenting concepts such as “pyramid-style parenting” and “elite-style parenting” influence your decision not to have two children? |
|  | Q35-5 | Does the fertility view focusing on emotional comfort through fulfilling family affection needs influence your decision to have two children? |
| Impact of challenges of childcare on actual fertility intention | Q37 | Does the issue of childcare before preschool affect your decision to have two children? |
|  | Q39 | Does the preschool drop-off and pick-up arrangement affect your decision to have two children? |
|  | Q41 | Does the primary school drop-off and pick-up arrangement affect your decision to have two children? |
| Impact of fertility policy on actual fertility intention | Q45 | Does the amount reimbursed under the current fertility policy affect your decision to have two children? |

| Supplementary Table 1 (continued) |  |  |
| --- | --- | --- |
| Dimensional indicators | Options | Item |
|  | Q46 | Does the level of support from your workplace, community, or village in terms of policies, regulations, and benefits for having a second child affect your decision to have two children? |
|  | Q47 | Does the current maternity leave policy affect your decision to have two children? |
| Impact of child upbringing on actual fertility intention | Q52-1 | Does the better physical and mental development of children affect your decision to have two children? |
|  | Q52-2 | Does a cooperative and supportive family environment influence your decision to have two children? |
|  | Q52-3 | Does the cultivation of children’s personality affect your decision to have two children? |
|  | Q52-4 | Does access to better family, school, and social educational resources affect your decision to have two children? |
| Impact of personal career development on actual fertility intention | Q53-1 | Does having two children affect your personal career development? |
|  | Q53-2 | Does having two children affect your interpersonal relationship development? |
|  | Q53-3 | Does having two children affect your quality of life? |

| Supplementary Table 2 Univariate analysis of factors influencing the willingness to have a second child among reproductive-aged women | | | | | |
| --- | --- | --- | --- | --- | --- |
| Characteristic | Category | Frequency (%) | Intention to Have a Second Child  [n (%) / (x̄±S.D.)] |  | *χ^2^*/*t* (*P*) |
|  |  |  | Unwilling | Willing |  |
| Participates | - | 2422(100.00) | 1259(51.98) | 1163(48.02) |  |
| Age | 18–29 | 793(32.74) | 515(64.94) | 278(35.06) | 93.08(<0.001)^1^ |
|  | 30–39 | 1029(42.49) | 506(49.17) | 523(50.83) |  |
|  | 40–45 | 600(24.77) | 238(39.67) | 362(60.33) |  |
| Residence | Urban areas | 1780(73.49) | 937(52.64) | 843(47.36) | 1.07(0.301) |
|  | Rural areas | 642(26.51) | 322(50.16) | 320(49.84) |  |
| Place of household registration | Jiaodong Peninsula | 329(13.58) | 209(63.53) | 120(36.47) | 71.90(<0.001)^2^ |
|  | Northwestern Shandong | 480(19.82) | 195(40.62) | 285(59.38) |  |
|  | Central Shandong | 945(39.02) | 554(58.62) | 391(41.38) |  |
|  | Southern Shandong | 668(27.58) | 301(45.06) | 367(54.94) |  |
| Educational level | Junior high school or below | 200(8.26) | 67(33.50) | 133(66.50) | 41.21(<0.001)^3^ |
|  | High school/Vocational High school/Technical secondary school | 305(12.59) | 137(44.92) | 168(55.08) |  |
|  | College / Bachelor's degree | 1678(69.28) | 929(55.36) | 749(44.64) |  |
|  | Postgraduate and above | 239(9.87) | 126(52.72) | 113(47.28) |  |
| Notes:  1: In any pairwise comparison of age, participants aged “30–39” had significantly higher willingness to have a second child than those aged “18–29”, participants aged “40–45” had significantly higher willingness to have a second child than those aged “18–29”, and participants aged “40–45” also had significantly higher willingness to have a second child than those aged “30–39” (*P* < 0.017 = 0.05 ÷ 3).  2: In any pairwise comparison of place of household registration, participants from “Northwestern Shandong” and “Southern Shandong” had significantly higher willingness to have a second child than those from “Jiaodong Peninsula”, participants from “Central Shandong” had significantly higher willingness to have a second child than those from “Northwestern Shandong” and participants from “Southern Shandong” had significantly higher willingness to have a second child than those from “Central Shandong” (*P* < 0.008 = 0.05 ÷ 6).  3: In any pairwise comparison of educational level, participants with “Junior high school or below” had significantly higher willingness to have a second child than those with “College/Bachelor's degree” or “Postgraduate and above”, and participants with “High school/Vocational/Technical secondary school” had significantly higher willingness than those with “College/Bachelor's degree” (*P*<0.008=0.05÷6). | | | | | |

| Supplementary Table 2 (continued) |  |  |  |  | |  |
| --- | --- | --- | --- | --- | --- | --- |
| Characteristic | Category | Frequency (%) | Intention to Have a Second Child  [n (%) / (x̄±S.D.)] | |  | *χ^2^*/*t* (*P*) |
|  |  |  | Unwilling | Willing | |  |
| Marital status | Unmarried | 587(24.24) | 421(71.72) | 166(28.28) | | 121.90(<0.001)^4^ |
|  | Married with no children | 206(8.51) | 100(48.54) | 106(51.46) | |  |
|  | Married with children | 1602(66.35) | 727(45.24) | 880(54.76) | |  |
|  | Widowed/divorced | 22(0.91) | 11(50.00) | 11(50.00) | |  |
| Only-child status | Yes | 580(23.95) | 345(59.48) | 235(40.52) | | 16.80(<0.001) |
|  | No | 1842(76.05) | 914(49.62) | 928(50.38) | |  |
| Type of original family | Nuclear family | 1213(50.08) | 700(57.71) | 513(42.29) | | 44.26(<0.001)^5^ |
|  | Backbone family | 993(41.00) | 444(44.71) | 549(55.29) | |  |
|  | Joint family | 123(5.08) | 56(45.53) | 67(54.47) | |  |
|  | Single parent family | 45(1.86) | 30(66.67) | 15(33.33) | |  |
|  | Reorganized family | 48(1.98) | 29(60.42) | 19(39.58) | |  |
| Notes:  4: In any pairwise comparison of marital status, participants with “Married with no children” and “Married with children” had significantly higher willingness to have a second child than those with “Unmarried” (*P*<0.008=0.05÷6).  5: In any pairwise comparison of type of original family, participants with “Backbone family” had significantly higher willingness to have a second child than those with “Nuclear family” (*P* < 0.005=0.05÷10). | | | | | | |

| Supplementary Table 2 (continued) |  |  |  |  | |  |
| --- | --- | --- | --- | --- | --- | --- |
| Characteristic | Category | Frequency (%) | Intention to Have a Second Child  [n (%) / (x̄±S.D.)] | |  | *χ^2^*/*t* (*P*) |
|  |  |  | Unwilling | Willing | |  |
| Occupation | Civil servant, public institution or state-owned enterprise personnel | 1163(48.02) | 578(49.70) | 585(50.30) | | 110.50(<0.001)^6^ |
|  | Foreign/private enterprise personnel | 149(6.15) | 86(57.72) | 63(42.28) | |  |
|  | Individual household | 97(4.00) | 43(44.33) | 54(55.67) | |  |
|  | Migrant worker | 68(2.81) | 27(39.71) | 41(60.29) | |  |
|  | Peasant | 174(7.18) | 64(36.78) | 110(63.22) | |  |
|  | Student | 336(13.87) | 255(75.89) | 81(24.11) | |  |
|  | Freelance work | 121(5.00) | 50(41.32) | 71(58.68) | |  |
|  | Other practitioners | 225(9.29) | 109(48.44) | 116(51.56) | |  |
|  | Unemployed | 89(3.67) | 47(52.81) | 42(47.19) | |  |
| Long-term migration status | Yes | 105(4.34) | 62(59.05) | 43(40.95) | | 1.91(0.167) |
|  | No | 2317(95.66) | 1197(51.66) | 1120(48.34) | |  |
| Household size | 1-2 | 176(7.27) | 104(59.09) | 72(40.91) | | 63.34(<0.001)^7^ |
|  | 3-4 | 1588(65.57) | 894(56.30) | 694(43.70) | |  |
|  | 5-6 | 619(25.56) | 237(38.29) | 382(61.71) | |  |
|  | ≥7 | 39(1.61) | 24(61.54) | 15(38.46) | |  |

Notes:

6: In any pairwise comparison of occupation, participants with “Civil servant, public institution or state-owned enterprise personnel” and “Individual household”, “Migrant worker”, “Peasant”, “Freelance work”, “Other practitioners” and “Unemployed” had significantly higher willingness to have a second child than those with “Student” (*P*<0.001=0.05÷36).

7: In any pairwise comparison of household size, participants with “5–6” members had significantly higher willingness to have a second child than those with “1-2” members and “3-4” members (*P*<0.008=0.05÷6).

| Supplementary Table 2 (continued) |  |  |  |  | |  |
| --- | --- | --- | --- | --- | --- | --- |
| Characteristic | Category | Frequency (%) | Intention to Have a Second Child  [n (%) / (x̄±S.D.)] | |  | *χ^2^*/*t* (*P*) |
|  |  |  | Unwilling | Willing | |  |
| Monthly household income | ≤6,000 RMB | 810(33.44) | 417(51.48) | 393(48.52) | | 1.61(0.806) |
|  | 6,001-10,000 RMB | 812(33.53) | 431(53.08) | 381(46.92) | |  |
|  | 10,001-15,000 RMB | 422(17.42) | 221(52.37) | 201(47.63) | |  |
|  | 15,001-20,000 RMB | 221(9.12) | 115(52.04) | 106(47.96) | |  |
|  | >20,000 RMB | 157(6.48) | 75(47.77) | 82(52.23) | |  |
|  | | | | | | |

| Supplementary Table 2 (continued) |  |  |  |  |  |
| --- | --- | --- | --- | --- | --- |
| Characteristic | Category | Frequency (%) | Intention to Have a Second Child  [n (%) / (x̄±S.D.)] |  | *χ^2^*/*t* (*P*) |
|  |  |  | Unwilling | Willing |  |
| Monthly household expenditure | ≤6,000 RMB | 1579(65.19) | 799(50.60) | 780(49.40) | 3.72(0.446) |
|  | 6,001-10,000 RMB | 662(27.33) | 361(54.53) | 301(45.47) |  |
|  | 10,001-15,000 RMB | 127(5.24) | 71(55.91) | 56(44.09) |  |
|  | 15,001-20,000 RMB | 33(1.36) | 17(51.52) | 16(48.48) |  |
|  | >20,000 RMB | 21(0.87) | 11(52.38) | 10(47.62) |  |
| Self-health status | Very healthy, no illness | 1006(41.54) | 489(48.61) | 517(51.39) | 19.17(<0.001)^8^ |
|  | Healthy, occasional minor illness | 1325(54.71) | 705(53.21) | 620(46.79) |  |
|  | Weak and prone to illness | 91(3.76) | 65(71.43) | 26(28.57) |  |
| Family health status | Very healthy, no illness | 889(36.71) | 417(46.91) | 472(53.09) | 16.59(<0.001)^9^ |
|  | Healthy, occasional minor illness | 1438(59.37) | 655(54.45) | 783(45.55) |  |
|  | Weak and prone to illness | 95(3.92) | 59(62.11) | 36(37.89) |  |
| Awareness of fertility policy | Not familiar | 483(19.94) | 305(63.15) | 178(36.85) | 46.89(<0.001)^10^ |
|  | Somewhat familiar | 890(36.75) | 393(44.16) | 497(55.84) |  |
|  | Quite familiar | 1049(43.31) | 561(53.48) | 488(46.52) |  |

Notes:

8: In any pairwise comparison of self-health status, participants with “Very healthy, no illness” and “Healthy, occasional minor illness” had significantly higher willingness to have a second child than those with “Weak and prone to illness” (*P*<0.017=0.05÷3).

9: In any pairwise comparison of family health status, participants with “Very healthy, no illness” and “Healthy, occasional minor illness” had significantly higher willingness to have a second child than those with “Weak and prone to illness” (*P*<0.017=0.05÷3).

10: In any pairwise comparison of awareness of fertility policy, participants with “Somewhat familiar” and “Quite familiar” had significantly higher willingness to have a second child than those with “Not familiar”, participants with “Somewhat familiar” had significantly higher willingness to have a second child than those with “Quite familiar” (*P*<0.017=0.05÷3).

| Supplementary Table 2 (continued) |  |  |  |  |  |
| --- | --- | --- | --- | --- | --- |
| Characteristic | Category | Frequency (%) | Intention to Have a Second Child  [n (%) / (x̄±S.D.)] |  | *χ^2^*/*t* (*P*) |
|  |  |  | Unwilling | Willing |  |
| Childcare before school enrollment | Primarily the mother | 457(28.05) | 281(61.49) | 176(38.51) | 7.31(0.199) |
|  | Both parents jointly | 177(10.87) | 105(59.32) | 72(40.68) |  |
|  | Paternal grandparents | 648(39.78) | 356(54.94) | 292(45.06) |  |
|  | Maternal grandparents | 277(17.00) | 168(60.65) | 109(39.35) |  |
|  | Hired nanny | 43(2.64) | 22(51.16) | 21(48.84) |  |
|  | Others | 27(1.66) | 18(66.67) | 9(33.33) |  |
| Actual fertility timing^#^ | Within one year | 90(4.20) | 25(27.78) | 65(72.22) | 44.30(<0.001)^11^ |
|  | One to two years | 1365(63.70) | 591(43.30) | 774(56.70) |  |
|  | Three to four years | 336(15.68) | 201(59.82) | 135(40.18) |  |
|  | Five years or more | 34(1.59) | 20(58.82) | 14(41.18) |  |
|  | Let nature take its course | 318(14.84) | 150(47.17) | 168(52.83) |  |
| Impact of household economic status on actual fertility intention | - | 2422 | 37.83±8.92 | 30.85±10.24 | 17.83(<0.001) |
| Impact of social and familial expectations on actual fertility intention | - | 2422 | 7.8±7.78 | 10.73±6.92 | -9.80(<0.001) |
| Impact of perceptions of fertility on actual fertility intention | - | 2422 | 10.59±5.84 | 11.15±5.27 | -2.50(0.013) |
| Notes:  11: In any pairwise comparison of actual fertility timing, participants with “Within one year” and “One to two years” had significantly higher willingness to have a second child than those with “Three to four years”, participants with “Let nature take its course” had significantly higher willingness to have a second child than those with “Three to four years” (*P*<0.005=0.05÷10). | | | | | |

| Supplementary Table 2 (continued) |  |  |  |  |  |
| --- | --- | --- | --- | --- | --- |
| Characteristic | Category | Frequency (%) | Intention to Have a Second Child  [n (%) / (x̄±S.D.)] |  | *χ^2^*/*t* (*P*) |
|  |  |  | Unwilling | Willing |  |
| Impact of challenges of childcare on actual fertility intention | - | 2422 | 11.16±3.55 | 9.15±3.70 | 13.65(<0.001) |
| Impact of fertility policy on actual fertility intention | - | 2422 | 16.92±4.38 | 14.45±5.32 | 12.37(<0.001) |
| Impact of child upbringing on actual fertility intention | - | 2422 | 11.91±3.23 | 9.47±3.61 | 17.45(<0.001) |
| Impact of personal career development on actual fertility intention | - | 2422 | 9.33±3.74 | 7.78±3.55 | 10.50(<0.001) |

Note: Actual childbirth timing refers to the duration between marriage and the birth of the first child.

| Supplementary Table 3 Collinearity test of independent variables | | |
| --- | --- | --- |
| Characteristic | VIF | Tolerance |
| Age | 1.376 | 0.727 |
| Place of household registration | 1.062 | 0.942 |
| Educational level | 1.396 | 0.716 |
| Marital status | 1.332 | 0.751 |
| Only-child status | 1.038 | 0.964 |
| Type of original family | 1.032 | 0.969 |
| Occupation | 1.104 | 0.906 |
| Household size | 1.088 | 0.919 |
| Self-health status | 1.240 | 0.806 |
| Family health status | 1.241 | 0.806 |
| Awareness of fertility policy | 1.033 | 0.968 |
| Actual fertility timing | 1.019 | 0.981 |
| Impact of household economic status on actual fertility intention | 1.470 | 0.680 |
| Impact of social and familial expectations on actual fertility intention | 1.745 | 0.573 |
| Impact of perceptions of fertility on actual fertility intention | 1.303 | 0.767 |
| Impact of challenges of childcare on actual fertility intention | 1.458 | 0.686 |

| Supplementary Table 3 (continued) |  |  |
| --- | --- | --- |
| Characteristic | VIF | Tolerance |
| Impact of fertility policy on actual fertility intention | 1.458 | 0.686 |
| Impact of child upbringing on actual fertility intention | 1.565 | 0.639 |
| Impact of personal career development on actual fertility intention | 1.480 | 0.676 |

Note：VIF：variance inflation factor

| Supplementary Table 4 The dominance analysis of factors influencing the willingness to have a second child | | | |
| --- | --- | --- | --- |
| Factor | Marginal Contribution | Standardized Weight | Importance Ranking |
| Actual fertility timing | 9.799 | 0.152 | 1 |
| Impact of household economic status on actual fertility intention | 7.613 | 0.118 | 2 |
| Awareness of fertility policy | 7.517 | 0.116 | 3 |
| Place of household registration | 7.413 | 0.115 | 4 |
| Impact of social and familial expectations on actual fertility intention | 5.612 | 0.087 | 5 |
| Marital Status | 5.566 | 0.086 | 6 |
| Impact of personal career development on actual fertility intention | 5.213 | 0.080 | 7 |
| Household size | 4.501 | 0.070 | 8 |
| Impact of challenges of childcare on actual fertility intention | 3.967 | 0.061 | 9 |
| Self-health status | 2.719 | 0.042 | 10 |
| Impact of perceptions of fertility on actual fertility intention | 2.440 | 0.038 | 11 |
| Only-child status | 2.335 | 0.036 | 12 |
